# *I expected to be pain free*: a qualitative study exploring athletes’ expectations and experiences of care received by sports chiropractors

**DOI:** 10.1186/s12998-022-00426-4

**Published:** 2022-05-02

**Authors:** Evan Eindhoven, Alex Lee, Peter Stilwell, Silvano Mior

**Affiliations:** 1 Department of Graduate Studies, Canadian Memorial Chiropractic College, 6100 Leslie St., Toronto, ON M2H 3J1 Canada; 2 School of Physical and Occupational Therapy, McGill University, Montreal, Canada; 3 Institute for Disability and Rehabilitation Research at, Ontario Tech University and Canadian Memorial Chiropractic College, Toronto, Canada; 4 Institute of Health Policy Management and Evaluation, University of Toronto, Toronto, Canada

**Keywords:** Athletes, Sport, Chiropractic, Qualitative study, Patient satisfaction

## Abstract

**Background:**

Knowledge about patient satisfaction and experience with care they receive can guide practitioners in establishing doctor-patient relationships and improve health outcomes. Although evidence suggests high patient satisfaction with chiropractic care in general, there is limited understanding of the expectations and experiences of athletes receiving sports chiropractic care.

**Objective:**

To explore the athletes’ expectations and experiences with care received from sports chiropractors, and their perceptions of relevant areas of future research.

**Methods:**

A qualitative study was conducted through an interpretivist lens exploring the perspectives of elite and competitive athletes receiving care from sports chiropractors in Canada. Participants were purposively recruited and interviewed until saturation was reached. Two research team members independently analyzed the interview transcripts using a conventional approach to content analysis. Content was inductively coded and discussed by the research team to generate categories.

**Results:**

We interviewed 18 participants between December 2018 and March 2020, 14 were national level athletes participating in sports ranging from paddling to combat sports. Reported reasons for seeking care included acute care, injury prevention, enhancing performance and maintenance care. Generated categories were organized under topics of experience with care, expectations of care, and research agenda. Participants experienced a variety of interventions, reassurance, varying treatment times, and reported positive impact on their athletic performance. They expected musculoskeletal assessment and treatment including at and beyond the injury site, symptom improvement, good communication and expertise from the chiropractor. Some participants suggested interpersonal and interprofessional communication can be improved, in particular the level of collaboration with other members of their health care team. Overall, participants reported a high level of trust and satisfaction with care received from sports chiropractors. From our participants’ perspective, suggested areas of research should focus on injury mechanics and prevention, impact of care on performance, and interprofessional collaboration.

**Conclusions:**

In general, participants were very satisfied with care. Overall, participants’ expectations and experiences aligned but changed over time. Addressing the findings of this study can be used to enhance the quality of care provided to athletes from sports chiropractors, as well as inform future research agendas. Further work assessing if athletes in other competitive levels have similar experiences and expectations is needed.

**Supplementary Information:**

The online version contains supplementary material available at 10.1186/s12998-022-00426-4.

## Background

The sport medical team is comprised of multidisciplinary professionals working together to serve the needs of the athlete. The team varies in composition and size depending on the event or level of play [1]. The team may include a chiropractor, physical therapist, athletic trainer, physician and osteopath, as well as strength and conditioning, sports nutrition, sports psychology, and other allied professionals with sports-related expertise [2]. Chiropractors are involved in the treatment of athletes ranging from amateur to professional levels. Stump et al. reported that 77% of athletic trainers in the National Football League (NFL) referred players to a chiropractor, and 31% of teams had one on staff [3]. Comparatively, it was reported that 75% of competitive Canadian taekwondo athletes and 85% of Canadian national team taekwondo athletes saw a chiropractor [4]. At the world games in 2013, 22% of Canadian athletes across all sports, and 18% across athletes from all countries participating at the world games sought chiropractic care [5]. Despite the level of utilization of chiropractic services among athletes, research exploring the experience of athletes undergoing such care has remained sparse.

Researchers have explored the role chiropractors play on sport medical teams [6–8]. Recently, Hostrup et al. explored the role of chiropractors in Danish football clubs suggesting clubs valued chiropractors on their medical team based on their expert disciplinary knowledge, improved diagnostic triage and increased treatment flexibility. However, in clubs without a chiropractor on their staff, they questioned the chiropractor’s expertise and considered the chiropractor as a technician or therapist [6]. The questioned sports expertise of chiropractors was also reported by Theberge, who explored the role of chiropractors among members of an Olympic sport medical team. They identified interprofessional boundary challenges threatening the inclusion of chiropractors on the healthcare team and suggested the main driving force for the inclusion of chiropractic care was the athletes’ expressed needs [7].

Despite the noted driving force of athletes for the inclusion of chiropractic care in sports, little is known of their experiences with care they receive. To the authors’ knowledge the only investigation of athlete satisfaction with chiropractic care was a report by Talmage et al. who reported high level of satisfaction among athletes interacting with chiropractors in a non-clinic sports setting, with communication noted as the most important factor [9]. While there is a paucity of research investigating the experience of athletes with chiropractic care in sports settings, the experience and expectations of athletes undergoing care has been studied amongst athletic therapists, with athletes reporting high levels of satisfaction with the care they received. These investigators found sport profile and gender influenced satisfaction rates, where lower profile sports and female athletes reported lower satisfaction. Additionally, empathy shown by athletic therapists through communication, advocacy and approachability was shown to positively affect treatment outcomes [10].

Outside of sports settings, investigations into the patient experience with chiropractic care in general practice suggests that satisfaction with care is influenced by communication, including ordering and relaying test results, the level of concern and empathy for, and how well things were explained to the patient [11–13]. It has also been shown that outcomes of care can only account for up to 57% of variance of patient satisfaction with 27% of this variance accounted through pain and effect on work, leaving 73% of patient improvement uncertain [12]. Experience and expectations of care by a health care provider suggests they contribute to satisfaction and treatment outcomes [9, 11–13].

However, athletes have been shown to differ from non-athlete populations in a variety of factors ranging from biological changes [14, 15] to differences in physical and social perceptions [16, 17]. Qualitative data suggest the contextual factors related to being an athlete impact how they perceive and experience an injury [18]. Additionally, the use of physical and complementary therapies has been reported to be a part of competitive athletes’ lifestyle practices [19]. It has been suggested that sports chiropractic care may differ from the care provided by general practice chiropractors, with self-report data revealing chiropractors with sports qualifications more often utilize a multimodal approach [20–23], frequently treat extremity conditions [21–23], and apply interventions with the intent to improve sports performance [23]. Despite these reports, clinical evidence of these defining characteristics of sports chiropractic practice has not been directly investigated, and it is not known how athletes perceive and experience the care provided by sports chiropractors.

Thus, to improve the care provided to athletes by sports chiropractors, further understanding of what athletes expect, what transpires during these care encounters, and their experience with sports chiropractic care is required. Therefore, the aim of this study was to explore the expectations and experiences with care provided by sports chiropractors by interviewing athletes currently competing at a high level and receiving care from a sports chiropractor.

## Methods

We conducted a qualitative interview study with an interpretivist lens [24] to explore the expectations and experiences of elite and competitive Canadian athletes receiving care from a sports chiropractor. For the purposes of our study, sports chiropractors were defined as chiropractors who have received specialized post graduate training and certification in assessing and treating athletes, such as members of the Royal College of Chiropractic Sports Sciences (Canada), the American Chiropractic Board of Sports Physicians or the International Federation of Sports Chiropractic. We also explored the athletes’ perspectives regarding relevant areas of future research. Situating our research within an interpretivist methodological paradigm allowed us to explore the subjective experiences and distinctive perspectives of participants and gain an understanding of the phenomena of interest in its unique social context [24].

### Recruitment

We used purposive sampling to recruit participants. Eligible participants included athletes under the care of a sports chiropractor, classified as competing at elite or competitive levels, and older than 16 years. We categorized participants by age, young (12–17), adult (18–35) and masters (35+), and by their highest level of competition, provincial, varsity or national [25]. Recruitment was assisted by a Study Advisory Committee comprised of members of the Royal College of Chiropractic Sports Sciences (Canada) Board of Directors who contacted sports chiropractors known to be working with participants meeting our inclusion criteria. All included sports chiropractors had completed a postgraduate education and fellowship examination with the Royal College of Chiropractic Sports Sciences (Canada) and were working in private practice. Some of the sports chiropractors had ties to sports teams and organizations where they had worked with the participants referred to the study, whereas others had worked with the participants solely in private practice. Sports chiropractors agreeing to assist contacted participants that met the inclusion criteria to determine their interest in participating. Participants agreeing to participate directly contacted the primary investigator (PI). Interested participants were provided study information and an informed consent form in advance of the interview. We also used snowball sampling to identify additional participants.

### Interview schedule and procedures

Semi-structured interviews were conducted by the PI via Skype (Skype Technologies, Microsoft, USA) or by phone, using an interview guide with open-ended questions. The semi-structured interview guide was developed in consultation with the Study Advisory Committee and informed by previous research [7], and pilot tested for comprehensibility prior to the start of the study (Appendix A). Recruitment and interviewing continued until data saturation was reached, as determined by the research team, whereby subsequent interviews added no new codes [26–28].

During the interviews, a second research team member served as a note-taker and when necessary, probed for clarification in areas of ambiguity. None of the researchers had prior knowledge of the study participants. All members of the research team were chiropractors, with two experienced in qualitative methods. Reflexivity, field notes, and team meetings were used throughout data collection and analysis to enhance the rigor and trustworthiness of the study [29]. The interviews ranged between 20 and 40 min in duration and were completed between December 2018 and March 2020. Interviews were audio-recorded, exported to an encrypted USB key, and transcribed verbatim by an experienced transcriptionist. All transcripts were reviewed for accuracy against the recorded sessions, errors corrected, and any potential identifying information removed prior to coding. Participants were invited to review their transcript and make any necessary edits to ensure their responses accurately reflected their intended responses. We did not conduct repeat interviews with any of the participants.

### Data analysis

Each transcript was independently coded by two members of the research team using a conventional approach to content analysis [30]. Transcript data were coded for both manifest and latent content, whereby codes were inductively created as interviews were analyzed [31]. After independently coding the transcript, paired researchers met to compare, discuss and resolve any discrepancies in coding. Codes were then collapsed into categories. Categories were refined by repeatedly comparing and verifying to the actual texts. A referent journal was kept to track the coding process, code definitions, timeline, and category development.

The coding structure and the related transcript reference data were entered into qualitative data analysis software (NVivo Pro Version 11.4.1 for Windows, QSR International (Americas) Inc., Burlington, MA, USA). Demographic data including the age, sex, current levels of participation, frequency of care, primary competitive sport, and participant location were descriptively analyzed. The qualitative analysis and subsequent reporting of the data was guided by the Consolidated Criteria for Reporting Qualitative Research (COREQ) [32].

### Ethics

Ethics approval was received from the Research Ethics Board of the Canadian Memorial Chiropractic College (# 1809X01). All participants were provided with an overview of the purpose of the study prior to participation and provided written consent when possible, and verbal when written consent was not able to be obtained.

## Results

### Participants

Nineteen participants referred from 11 sports chiropractors, with a maximum of 4 participants from any one chiropractor, agreed to be interviewed, and one withdrew prior to scheduling the interview. Interviewed participants included 10 females and 8 males, with ages ranging between 16 and 68 years. They competed in various sports from varsity (n = 2) to provincial (n = 2) to national levels (n = 14) (Table 1).

**Table 1:** Participant demographics

Subject number	Sex	Sport	Age range	Level of competition
1	Female	Track and field	Adult	National
2	Male	Weight lifting	Master	Provincial
3	Male	Lacrosse	Adult	Varsity
4	Female	Gymnastics	Young	National
5	Female	Track and field	Master	National
6	Female	Hockey	Adult	National
7	Male	Baseball	Adult	Varsity
8	Male	Track and field	Adult	National
9	Female	Paddling	Adult	National
10	Female	Track and field	Adult	National
11	Female	Paddling	Master	National
12	Male	Skiing	Adult	National
13	Female	Combat sports	Adult	National
14	Male	Skiing	Adult	National
15	Male	Combat sports	Master	National
16	Female	Combat sports	Adult	Provincial
17	Male	Football	Adult	National
18	Female	Combat sports	Adult	National

All interviews were conducted between December 17, 2018 and March 19, 2020. Data saturation was deemed to be reached by interview 15. All of the participants interviewed reviewed their transcripts, but none made edits to their transcript.

Results were organized under three overarching areas focused on the study’s aim. These areas included athlete expectations of sports chiropractors, athlete experiences with sports chiropractors, and future research direction. Under the area athlete expectations of sports chiropractors, the related categories reflected the participants’ views of their expectations when seeking care and during their chiropractic encounter. In the area of athlete experiences with the care received, categories included “inside the visit” (experiences within the sports chiropractic encounter), referral source, collaboration, satisfaction, and trust. In the third area, participants expressed their perspectives of the recommended research direction for the sports chiropractic field, namely injury mechanics and prevention, and performance.

## “*I expected to be pain free*”: athlete expectations of sports chiropractors

Participants entering care had expectations of their sports chiropractic visit encounter. Although some differences existed between participants based on their prior health care experiences, overall considerable overlap between participants was noted. These expectations were organized into two broad categories namely, expectations upon entering care (Seeking Care), and during their encounter (The Encounter).

### Seeking care

Most participants sought care from a sports chiropractor primarily for an injury incurred while competing or training. They expected the chiropractor would manage their injuries, alleviate symptoms, and facilitate return to sport or training at their desired level.

*“Pain relief and recovery, and both of those things happened so I was able to proceed with the new sport that I was going to love very quickly.”* S11

Other participants sought care to help with injury prevention, reduce the potential of their injury progressing to a point that it would interfere with their training and competition, or improve performance. This preventative perspective is similar to the concept of maintenance care or pre-habilitation, where a patient seeks care to maintain their current health status or prevent future injuries or regression during their rehabilitation [13]. The idea of maintenance care is often discussed in chiropractic practice [33], and in our study sample it appeared to have some overlap with performance care, for example,

*“Yeah, it was for performance and just regular maintenance.”* S14.

*“I had a lot of injuries so I was hoping to learn some pre-rehab, how to prevent injuries, but then also I was hoping to have him be the guy who fine-tuned me before races after having the experience at nationals going from, I don’t know if I will be able to run to being able to win... I mean that speaks for itself. So, a little bit of pre-rehab and then also just like maintenance and just become more durable.”* S8

Participants also expected to receive self-care strategies that would assist in managing their injuries and symptoms. Many participants reported the care and self-management strategies they received enabled them to continue to train at their highest level without interference from minor aches and pains.

### The encounter

Participants with no prior sports chiropractic experience were surprised at the level of interpersonal interaction, which differed from their experience with physiotherapists and general practice chiropractors. These participants assumed the care provided by a sports chiropractor would primarily focus on spinal care and expected less hands-on or intensive care than they experienced. This notion of focused spine care seemed grounded in their preconceived belief that sports chiropractic care would be similar to care received from other therapists who focused primarily on exercise therapy, non-sports chiropractors or what they heard in the media. For example:

*“I guess initially when I went to see the chiropractor, I guess there was an expectation that they would just kind of deal with my spine* (laugh) *and stuff like that, but I also feel like as I started seeing more chiropractors, I was exposed to chiropractors that had a larger scope of practice.”* S10

Some participants also expected the visit to be brief, focussed on joint and spine assessment, and treatment primarily involving high velocity, low amplitude (HVLA) spinal manipulation. Instead, participants expressed surprise when their encounter included a comprehensive physical examination that assessed bodily regions beyond their injury location, detailed communication of the problem, and a custom-tailored treatment plan addressing the specific participant’s concerns and needs. Many participants reported only experiencing care from a sport medical team and were unaware of what sports chiropractic care could add to their care.

*“I was expecting just to get like adjusted. I feel the reputation chiropractors have is just like straight up crack your back and leave but I got so much more.”* S8

*“I didn’t really have any pre-conceived notions honestly* (laugh) *and I was just kind of expecting normal physiotherapy, kind of what we had gotten used to over the years…. active release, needles, like general preventative work and assessing issues and that kind of stuff, which is essentially what she does. She just has a more balanced approach to it all.”* S14

Participants had different expectations of their clinical encounter depending upon their prior therapeutic experiences but appreciated the diversity in the style used by the sports chiropractor. However, as care progressed and their condition improved, participants’ expectations shifted, such that some participants expected more during their visits than just a resolution of pain. They expected the sports chiropractor perform more in-depth assessment examining more than just the injured or painful area and expand their plan of management to include performance enhancement, injury prevention, or in some cases, psychological re-assurance.

*“When I walked in, I expected only just to be pain free, but after like my first two visits, I was like, I won’t just come out pain free but I will come out of it better than I originally was before I was hurt.”* S7

## *“See the whole picture”:* athlete experiences with sports chiropractors

Although differences existed between the experiences shared by each participant during the visit encounter with the sports chiropractor, most were similarly categorized. Specifically, they reported similar treatment interventions received and discussed varying visit durations and interprofessional collaborations. Participants described their high level of satisfaction with the care received from the sports chiropractor and their opinions how interprofessional interactions could improve.

### Inside the visit

Participants described the typical visit encounter with their sports chiropractor as involving some level of assessment and treatment. They described their assessment as typically involving the examination of multiple areas of the body, rather than focused around the area of complaint. The thorough assessment created a perception that the sports chiropractor was concerned about their “*whole picture*”; while also considering the other reasons that were potentially causing or contributing to their problem, including sport technique, injuries in other areas, and psychological impact of the injury.

*“…at some point early on, she took each of us for a full assessment and just wanted to see the whole picture. So, it wasn’t like I just had some little thing nagging me and I saw her about that. Like she wanted to do the whole assessment, get the big picture and really be able to give me the best treatment I could get and not just focus on one little thing.”* S12

Participants described their plan of management as including different interventions. These included HVLA joint manipulation, interventions targeting joints, soft tissue therapy, passive modalities, acupuncture, and rehabilitative exercises. Treatment plans were described as being customized for each participant, but typically the core interventions included manual therapy and rehabilitation exercises, supplemented with additional therapeutic modalities as needed.

The participants associated the duration of their visit with perceived thoroughness of the assessment, depth of communication, and appropriateness of applied interventions. Visit duration reportedly ranged between 5 and 90 min. Most participants appreciated and were grateful for the longer visit durations, enabling them to discuss their concerns and communicate what they were experiencing. This added time ensured each of their issues were voiced, explained and addressed by the sports chiropractor. As noted by one participant, they felt “*fortunate for* (chiropractor) *to be able to spend all that time on me” S1,* knowing that other participants and patients of other chiropractors may not be receiving the same amount of time or attention.

However, some participants experienced a much shorter visit duration, and considered this an area for improvement by the profession. This highlights the reported office-to-office variation in visit duration in chiropractic offices. Despite feeling satisfied with the care they received, some participants suggested their interaction with the sports chiropractor could be improved with longer visit duration or the ability to book additional treatments or time slots with them.

*“Never have I been there 30 minutes. We could be in and out in 5 minutes depending on your injury or we could be there for 15-20 minutes. They are usually quick depending on your injury.”* S4

*“Yes. The only down-side to this is that most of my appointments are like 15 minutes, so even with that 15 minutes I feel like I need to make an appointment back-to-back, so I am able to address everything. That would be the only thing that is kind of annoying.”* S7

### Referrals

The majority of participants were referred to a sports chiropractor from another athlete, either on the same team or from a similar sport. This referral from a trusted source added credibility to the chiropractor.

*“A previous athlete that had seen him. I tend to kind of go off of another high-performance athlete who has seen someone and would recommend them and then I will give them a try.”* S10

Others indicated they were referred by their coaches or parents. In some cases, the strength of the referral encouraged the participant to return for care despite a bad initial experience with the chiropractor. As reflected by this participant, despite a challenging first encounter, they continued with care and eventually built a strong relationship with their sports chiropractor:

*“I think it was the strength of the referral, knowing this person had been on* (international team)*, I’d give him another chance. I wouldn’t have given him many more chances* (laugh)*.”* S5

### Interprofessional collaboration

Participants described varying degrees of interprofessional collaboration and communication between their sports chiropractors and other health care providers. They perceived higher levels of interprofessional collaboration if they were managed by multiple practitioners in the same facility or if their care occurred within a sport organization.

*“He writes it in her file and being as they are in the same office; they are able to talk back and forth. So, the physio can read the chiropractors notes and vice versa. So that is super helpful.”* S4

Many participants reported receiving care from several health care providers, but not all participants reported there was any collaboration between the health care providers within their care network. The varying and often limited communication between healthcare providers was a common topic among the participants, especially when liaising with providers from other offices, especially medical doctors. Participants frequently had to advocate for themselves and noted this as an area that could be improved within sports chiropractic care.

*“… have another S&C* (Strength and Conditioning) *Coach they were in communication with each other because that one has known me for years, so he was kind of giving him insight into me. But in terms of like across treatment and like my massage therapist and whatnot, not really. I feel like in general I am kind of the middle person* (laugh) *and so I communicate and am directing everything for myself.”* S10

### Satisfaction

All participants interviewed reported being satisfied with the care they received from their sports chiropractor. Participant’s satisfaction appeared related to the chiropractors’ clinical expertise, related sport knowledge, good interpersonal communication skills, and their positive outcomes from care.

*“I am extremely satisfied and I know there are other chiropractors in the office that would give very good treatment as well, but I am so comfortable with Dr. X and he knows my injuries so well that I like going back to him.”* S11

Although all participants were satisfied with care, they reported there was room for improvement in the care they received. Based on the responses of participants in our study, communication plays a critical role in creating satisfaction with care.

*“The only thing I would kind of like to see a little bit more of is just how he does it, I guess. He kind of explains, oh like this is what I am doing, but it is more to know like I know what you are saying you are going to do but I have no idea what that means essentially. What you are adjusting and how you adjust it?”* S7

### Trust

All participants noted they trusted their sports chiropractor and were comfortable discussing all aspects of their training and personal wellbeing. This established trust enabled the participant to feel at ease to discuss their experiences and feel confident the sports chiropractor would be able to help them. Some participants noted that they did not have this same level of trust when seeking care from non-sports chiropractors or other medical practitioners, and consequently felt they had a less favourable clinical experience.

*“The injuries were overcome, and I was able to get back to training, my pain is gone, all those things were successful. So, you know, why would I go elsewhere? I have to drive 20 minutes on the highway to* (location) *to get to see him but that is how much I trust in his ability to help me.”* S11

*“I mean he is the professional here so there is a lot of trust in that relationship. So for him to be able to say hey this seems kind of tight or this seems like it is not working like I want it to and for him to go in and try to fix it I think is great.”* S6

### Performance

Most participants believed the care they received positively impacted their physical performance. In addition to the physical needs of the participant, they also felt comfortable voicing their psychological and emotional concerns. They also felt this improved their psychological aspect of their performance by being reassured their fears and beliefs about their physical condition would enable them to achieve peak performance.

*“I mean, I have had you know some other injuries where they are more serious than others but usually it is just yeah getting peak performance. Usually when I am getting ready for a contest, I see him more often because I am lifting heavier. I notice that makes a difference.”* S2

*“So sometimes I may hold myself back just so that I don’t hurt myself, but then I won’t perform at the level that I am supposed to be performing at. But if I am being treated, I will feel a lot more confident and be able to kind of like, risk my body a little bit more, and in turn play better, perform better because I know that when I get off the field, I will be getting treatment to be able to play again the next week and be okay. If I don’t feel like I will be getting the right treatment to be able to perform the next week, then I might be a little bit more hesitant in terms of how I play. Does that make sense?”* S17

Performance may be influenced at the mental level or with improved efficiency and confidence of the movement performed after an intervention [34].

*“I would definitely say there is an all-inclusive package of things that make you feel good when you perform and having a body that is ready to go and doesn’t have any pain is a contributing factor to pulling faster.”* S9

## “*Finding thousandths is not insignificant*”: suggestions for a research agenda

Participants were questioned about their opinion of the type and nature of research they felt important in developing a research agenda in sports chiropractic, and the types of research they think would best benefit the athletic community in the future. Participants were interested in seeing research performed in several areas they considered important, namely injury mechanics and prevention, and care enhancing sport performance.

### Injury mechanics and prevention

Many participants suggested research be conducted to learn about the mechanics of how injuries occurred and what they could do to prevent them from happening. Injuries appeared to be a major frustration for participants as they impact their training intensity and prevent them from competing.

*“I don’t know if I am just a mess, but I can have a great week of training and then like take the weekend off and then literally just do walking and like have a twinge in my ankle or my knee or something, like what is that? Is that preventable or just me breaking down over time? But anyway, yeah I would say injury prevention needs to be studied more in my opinion.”* S17

*“I think possibly mechanism of injury like in sport. Just to better educate the athletes. I mean even myself I don’t know a lot about like how posture can affect your back when you are lifting, stuff like that. Something I have learned over the last little while but something that definitely could have helped me before I started having the issues. That if I was more aware of that it could have prevented some problems.”* S3

Participants wanted to understand how and why injuries happen, and what they could do to prevent them. They felt research assessing changes in training techniques or strategies to prevent minor day-to-day strains that limit their ability to operate at their best is important. Additionally, participants wanted to know when training can become too much and when it is no longer helpful for them to keep pushing at their current pace. For participants, balancing their training volumes appeared just as important to study as mechanics to understand how to prevent injuries. Many participants push themselves very hard and for some this amount of load may take away from their ability to perform.

*“So, if I am wrestling and a guy picks me up and throws me on my head obviously my neck is going to be sore for a few days. I am not talking about that. I am talking about the gruelling work-outs that we do day in and day out and at what point are we getting to a point where we are over training and our bodies are no longer building muscle and burning fat and we are just breaking down, break down of tendons and joints and stuff.”* S18

### Performance enhancement

Regarding sport performance research, participants suggested how to measure performance. In their opinion, improved performance may not always be measurable using traditional methods. Participants were particularly interested in research that would contribute to understanding how sports chiropractic could enhance their performance. As athletes, they want to be able to optimize their training and competition results because even small gains can lead to important outcomes.

*“I guess for me it would be the effects of chiropractic treatment on performance in terms of like I think chiropractic treatment, people often think of it in terms of like injuries, so kind of maybe showing how that work can be used [to] find the extra 3% for performance type of thing if that makes any sense.”* S10

Researchers may need to modify their methods and outcomes to better capture the various dimensions of performance enhancements reported by athletes. Research assessing the effect of interventions on performance has produced conflicting results or small changes in performance [39]. This becomes a challenge in sports where small measured objective changes may be important to the athlete, particularly for those in sprinting, running and power sports. One participant suggested that a change of a thousandth of a second may be significant to them.

*“Literally I mean like finding thousandths is not insignificant.”* S10.

Participants suggested other metrics that could potentially be used to measure changes in performance, such as range of motion, speed, balance and function. These metrics were considered important to the participant, specifically in understanding how they may directly translate to improved performance during competition.

*”Well, I think depending, so for example for something like a side kick probably you would be looking at range of motion and the speed and power. You know probably for most of the, I just find for me personally, an adjustment usually means to me range of motion and balance and would be the most applicable for me.”* S16

Participants also suggested that it may not just be the ability to perform a movement, but to be able to perform the movement with better balance or without pain. These may directly translate into the athlete’s ability across an event or competition and allow them to compete at their best. Participants want research focused on different ways of measuring sport performance, so that they may be able to learn more about these aspects and how they can integrate such findings into their routines.

*“Well like I bend but sometimes it is a little bit sore because I am so tight, so when I go and get an adjustment it just sort of relaxes everything so it doesn’t hurt at all to bend in half.”* S4

## Discussion

To our knowledge, this is the first qualitative study exploring the expectations and experiences of Canadian athletes undergoing sports chiropractic care. Our results suggest that athletes seek care from sports chiropractors for injury resolution, to prevent injuries, and to enhance their sport performance. The participants in our study reported a high level of trust and satisfaction with their sports chiropractor, expect good communication, full body assessments and treatment, and care that allows them to continue to perform at a high level.

The participants interviewed in our study initially sought sports chiropractic care for an injury; however, as care progressed the participant’s expectations for care included injury prevention and performance enhancement. Our findings are similar to previous work where the expectations of athletes of sports medicine professionals is primarily for injury care [18, 34, 36]. Similarly, elite Irish rowers seeking care from sports physiotherapists, most frequently expected injury treatment, followed by injury prevention and then lastly for performance enhancement [34]. Studies evaluating patient expectations with general chiropractic and complimentary and alternative therapies have identified an initial expectation of pain relief [35, 37, 38] and as care progressed, the expectations of the patients shifted towards improvement of function [38], self-care [37] and whole person healing [38]. The shifting of expectations of athletes from injury care to injury prevention and performance enhancement may represent a natural care evolution from the urgent need to treat an injury, to preventing an injury, and eventually shifting to improve the athlete’s well-being to enhance their performance.

For some participants, their initial perception of sports chiropractic care focused on spinal care and spinal manipulative therapy, but as they experienced care, they were surprised at the range of interventions provided during the encounter. Many participants described that their sports chiropractor took the time to identify the cause of their problem, which sometimes involved addressing their sports technique or injury mechanism, and often involved evaluating and treating areas distant to the injured site to improve their overall body mechanics. Our qualitative data support previous self-report surveys characterizing sports chiropractic practice that reported the frequent use of multi-modal care interventions [20–23], the treatment of the extremities [21–23], and treatment intentions to improve sport performance [22, 23]. While providing musculoskeletal assessment and treatment including at and beyond the injury site, this approach to care is not unique to sports chiropractors. A functional full body approach utilizing multi-modal care with the intent to improve function and performance may be a characteristic of sports chiropractic practice. Future practice-based research can provide insight on the defining features of sports chiropractic practice.

Many participants in our study reported that the care they received had a positive impact on their performance. A recent systematic review found that there was no significant effect of spinal manipulation on sport-specific measures of performance but did identify small changes in parameters where the significance of the change in performance was uncertain [39]. Another systematic review found low quality evidence that spinal manipulation improved surrogate measures of sport performance, such as hip extension velocity during running and kicking speed in soccer players but drew no firm conclusions on its influences on sport performance [40]. Similarly, a meta-analysis of studies evaluating the effect of massage on performance recovery found conflicting outcomes [41]. The participants in our study reported that they felt that care allowed them to recover better from training and move more efficiently, which they attribute to an improvement in their performance. Considering the many determinants of athletic performance, studying the effect of a single intervention on a specific performance metric may not fully capture the overall treatment encounter effect on performance experienced by athletes. It is possible that the perceived enhanced performance by the athlete may be a consequence of the interaction of the full encounter, including the intervention(s), the psychological effects of receiving treatment, facilitating the athlete’s pre-competition routine, social support to the athlete, and reassurance. In a qualitative study of Olympic, Paralympic and world champion athletes, Burns et al. [19] identified four themes that elite athletes attribute to their performance success: psychological attributes, performance strategies, interpersonal relationships and lifestyle practices, with all athletes attributing most of their success to psychological rather than physical factors. They mentioned their reliance on faith, routines or rituals; use of physical therapies and recovery; sports healthcare providers as part of their interpersonal support team; and the utilization of complementary therapies as part of their lifestyle practices. These investigators posit that performance enhancement is likely to occur at the intersection of psychological prowess, interpersonal support, effective performance strategies and lifestyle. It is possible that the sports chiropractic encounter may confer such benefits to each of these domains as noted by the participants in our study. Future work should investigate the impact of the sports chiropractic encounter on these domains, and if they contribute to athletic performance success.

Furthermore, the participants we interviewed referred to the importance of their sports chiropractor providing psychological and social support in their care. This suggests sports chiropractors consider the “whole athlete”, with attention to the contextual factors operating within a performance-driven environment [18], where issues affecting their performance, or even worse, leading to time-loss from sport, can cause considerable psychosocial distress [18, 19, 42]. For example, Arvinen-Barrow et al. reported that athletes expected to be injured, and they recognized that injury is “part of the job” [36]; however, the athletes mentioned that injuries are a main source of frustration for them as it often affects performance and can lead to time-loss from sport [18, 42]. In a qualitative study examining how elite athletes, coaches and physiotherapists perceive a sports injury, Bolling et al. [18] recommended that sports injuries for athletes should not be viewed as a health condition in itself, but rather as an interaction between physical damage and contextual factors. In a proposed framework to operationalize a sports injury for athletes, Timpka et al. [43] applied the International Classification of Functioning, Disability and Health (ICF) by the World Health Organization to athletic injuries. In the ICF framework, these authors recommended the use of the terms “sports injury and disease” as a diagnosis, “sports trauma and illness” to represent its impact on body functions and structures, and “sports incapacity and sickness” to represent the impact of the injury to an athlete’s sports participation. The ICF emphasizes the biopsychosocial model that recognizes the importance of environmental and personal contextual factors, which for athletes can include a lifestyle committed to training and working in a performance-driven environment. As reported by some participants in our study, the sports chiropractor provided psychosocial support during the patient encounter, thereby creating a therapeutic alliance with the athlete and optimizing contextualized care.

Although all participants in our study were satisfied with the care they received, some reported lower satisfaction with chiropractors they had previously seen who did not specialize in sport. This dissatisfaction appeared related to perceived differences in skills and style of treatment and/or lack of understanding of the athlete’s unique context. It has been reported that athletes prefer that their healthcare providers understand and share their sport culture [46]. Athletes report that practitioners with experience in their sport, as a former athlete or as a practitioner, are considered to better relate and empathize with their situation [47, 48]. It is possible that the appreciation for the athlete’s sport and their athletic culture may be a defining feature between sports chiropractors and general chiropractors. In addition to the sports-specific injury knowledge sports chiropractors possess, their advanced sports training may also provide them with unique knowledge of the athletic context, preparing them to better care for the athletes they treat.

From our interviews, participants reported on areas where they believed their care could be improved, notably the duration of their visit and interprofessional communication. We noted discrepancies in time spent by the participant with the sports chiropractor, with appointment durations ranging from 5 to 90 min. Our findings support similar findings noted in general chiropractic practices where visit lengths varied, and patients were typically more satisfied with a longer treatment duration [49]. It is likely that the preference for longer visit durations is not unique to athletes; however, longer duration visits may be better suited to encounters where a multi-site MSK assessment and treatment and contextualized healthcare approach can be better conducted.

Despite the participants’ satisfaction with communication with their chiropractor, the reporting of inter-professional communication amongst a participant’s healthcare providers varied. In our study, many participants were likely to report improved interprofessional communication when their healthcare team was co-located or part of a sport organization’s provider network. A lack of communication and collaboration between providers can negatively impact the quality and efficiency of care provided to athletes, as well as lead to communication barriers and role understanding with other professionals in an interdisciplinary framework [50]. As noted by Theberge, some professionals do not understand the role of the chiropractor in the sport medical team [7, 8]. Interprofessional teams and co-management of patients may help decrease the fragmentation of care (care without central coordination) and enhance both continuity and quality of care [50]. By enhancing communication, sports chiropractors may be able to better care for athletes while enhancing the understanding of the chiropractic role on an interprofessional team.

We also explored the expectations athletes have of sports chiropractors in contributing to research, as well as their opinion of research priorities. Participants described they wanted research that focused on understanding the mechanisms behind how an injury occurs, how to prevent injuries, and how care can aid in enhancing their performance. These suggested research priorities are in line with our findings that athletes seek care from sports chiropractors for injury resolution, to prevent injuries, and to enhance their sport performance. These findings can be used to inform research agenda development to strengthen the value of future research performed in the sports chiropractic field.

Our study has strengths and limitations, with a strength being that we interviewed athletes across different competitive levels, from locations across Canada, and from a variety of sports backgrounds. It should be noted that our study’s aim was to investigate the expectations and experiences of Canadian athletes undergoing sports chiropractic care, so our findings may not be generalizable to athletes receiving care from sports chiropractors from other countries due to potential cultural differences. In fact, the expectations of athletes from the United Kingdom receiving care from athletic trainers have been shown to differ from those from the United States of America [51]. Future research can investigate whether athletes’ expectations and experiences of sports chiropractors are different in other countries. Additionally, all researchers analyzing the data were chiropractors. As with all interpretive qualitative studies, the nature of the data analysis may have yielded different findings if analyzed from a different perspective. Another limitation was sports chiropractors were enlisted to assist in recruitment by identifying and referring athletes to the study team, which may have excluded athletes who may have been dissatisfied with care or had less favourable outcomes. Future studies could reduce this bias by recruiting athletes directly from athlete and/or sports organizations.

## Conclusion

In conclusion, the elite and competitive athletes interviewed in this study who were under care of a sports chiropractor in Canada expect injury resolution and/or prevention. They expected to have improved outcomes and desired longer treatment sessions. They experienced care involving musculoskeletal assessment and treatment including at and beyond the injury site, use of diverse interventions, and good interpersonal communication that contributed to high levels of satisfaction. However, some participants suggested interpersonal and interprofessional communication could be improved, particularly at the level of collaboration with other members of their health care team. The participants suggested that research should assess the effects of treatment on performance, injury mechanics and injury prevention.

## Supplementary Information

**Additional file 1.** Consolidated Criteria for Reporting Qualitative Studies (COREQ) Checklist.

**Additional file 2.** Appendix A: Interview Guide.

## Data Availability

Study data and materials are being securely stored by the authors, and are not readily accessible to the public consistent with patient informed consent and as approved by the Research Ethics Board.
