# Supplementary material for: I expected to be pain free: a qualitative study exploring athletes’ expectations and experiences of care received by sports chiropractors
Source: Chiropr Man Therap. 2022 May 2;30:21. doi: 10.1186/s12998-022-00426-4 (PMC9059405; doi:10.1186/s12998-022-00426-4)
Supplement: Supplementary file 1 — Additional file 1. Consolidated Criteria for Reporting Qualitative Studies (COREQ) Checklist. [file 12998_2022_426_MOESM1_ESM.docx]

Consolidated Criteria for Reporting Qualitative Studies (COREQ) Checklist

| **Domain 1: Research Team and Reflexivity** | | |  |
| --- | --- | --- | --- |
| **Personal Characteristics** | | **Page Description Found and Comment** | **Pg No Reported** |
| Interviewer/ Facilitator | Who conducted interviews? | Interviews were conducted by all members of the research team pending researcher availability | Pgs 6 and 7 |
| Credentials | What were the researchers’ credentials? | Credentials of authors include:  DC – all,  FRCCSS(C) – Canadian sport designation  PhD - two | Title page |
| Occupation | What were the researchers’ occupation(s) at time of study? | Chiropractors and Chiropractic Researchers | Pg 7 |
| Gender | Researcher genders? | Male |  |
| Experience/ Training | Experience and training of researchers? | Chiropractors and two with experienced in qualitative research methods | Pg 7 |
| **Relationship with Participants** | | |  |
| Relationship Established | Researcher established relationship with participants before start of study? | Members of the research team had no formal relationships with research participants before the study commenced | Pg 7 |
| Participant Knowledge of Interviewer | What did participants know about researchers? | Participants were made aware of researcher credentials, affiliations, and aims of the study prior to providing consent; otherwise, did not know the researchers. |  |
| Interviewer Characteristics | What researcher characteristics reported to participants? | All credentials and affiliations were reported to participants prior to providing consent and performing the interview | Informed consent (available on request) |
| **Domain 2: Study Design** | | |  |
| **Theoretical Framework** | | |  |
| Methodological Orientation & Theory | Methodological orientation underpins study? | Qualitative study from an Interpretivist lens | Pg 6 |
| **Participant Selection** | | |  |
| Sampling | How were participants selected? | Purposeful and snowball sampling | Pg 6 |
| Method of Approach | How were participants approached? | Participants were approached by their Chiropractor and asked if they had interest in participating in the study | Pg 6 |
| Sample Size | How many participants were in the study? | 19 athletes were approached, one withdrew, thus leaving 18 for analysis | Pg 9 |
| Non-participation | How many people refused to participate or dropped out? Reasons? | 1 – unable to find a time to schedule the interview that worked for both researchers and the participant | Pg 9 |
| **Setting** | | |  |
| Setting of Data Collection | Where was the data collected? | Data collected either by phone or via Skype, and recorded using voice recorders and voice recording software | Pg 6 |
| Presence of Non-participants | Was anyone else present besides the participants and researchers? | No | Pg 7 |
| Sample Description | What are the important characteristics of the sample? | Canadian Athletes undergoing care from a Sport Chiropractor | Pgs 6 and 9 |
| **Data Collection** | | |  |
| Interview Guide | Were questions, prompts, guides provided by the authors? Was it pilot tested? | Yes, the interview guide was pilot tested.  The interview guide is available upon request. | Pg 7 |
| Repeat Interviews | Were repeat interviews made? | No | Pg 7 |
| Audio/visual Recording | Did research use audio or visual recording to collect the data? | Audio | Pg 7 |
| Field notes | Were field notes made during and/or after the interview? | Yes, filed notes were made by researchers during the interview and during analysis of data | Pg 7 |
| Duration | What was the duration of the interviews? | Interview time varied but did not exceed an hour per participant | Pg 7 |
| Data Saturation | Was data saturation discussed? | Yes | Pgs 7 and 10 |
| Transcripts Returned | Were transcripts returned to participants for comment and/or correction? | Yes, they were returned for correction | Pgs 7 and 10 |
| **Domain 3: Data Analysis and Findings** | | |  |
| **Data Analysis** | | |  |
| Number of Data Coders | How many data coders coded the data? | 4 | Pg 7 |
| Description of Coding Tree | Did authors provide a description of the coding tree? | Yes, a description of the coding tree was provided in the Results | Pg 10 |
| Derivation of Themes | Were themes identified in advance or derived from the data? | Themes were derived from the data. | Pgs 7-8 |
| Software | What software, if applicable, was used to manage the data? | NVivo Pro Version 11.4.1 for Windows, QSR International (Americas) Inc., Burlington, MA, USA | Pg 8 |
| Participant Checking | Did participants provide feedback on the findings? | No, they were asked to provide feedback on their transcribed interview | Pgs 7 and 10 |
| **Reporting** | | |  |
| Quotations Presented | Were participant quotations presented to illustrate themes / findings? Was each quotation identified? | Yes – quotes were identified to an anonymized participant | Pgs 10 to 22 |
| Data/Findings Consistent | Was there consistency between the data presented and the findings? | Yes, | Pgs 7 to 8 |
| Clarity Major Themes | Were major themes clearly presented in the findings? | Yes, each overarching area or theme was noted, described and bolded in the text. | Pg 10, and pgs 10 to 22 |
| Clarity Minor Themes | Is there a description of diverse cases or discussion of minor themes? | Yes, discrepant perspectives were noted and discussed. |  |

<https://academic.oup.com/intqhc/article/19/6/349/1791966/Consolidated-criteria-for-reporting-qualitative>
