# Supplementary material for: I expected to be pain free: a qualitative study exploring athletes’ expectations and experiences of care received by sports chiropractors
Source: Chiropr Man Therap. 2022 May 2;30:21. doi: 10.1186/s12998-022-00426-4 (PMC9059405; doi:10.1186/s12998-022-00426-4)
Supplement: Supplementary file 2 — Additional file 2. Appendix A: Interview Guide. [file 12998_2022_426_MOESM2_ESM.docx]

## Appendix A: Interview Guide

1. Can you please tell me about your involvement in sports?
2. Why did you decide to attend your current chiropractor? How long have you been attending your current chiropractor?
3. What if any was your expectation prior to seeing the chiropractor?
4. Were your expectations met? Did your expectations change over time and why?
5. Did your experience with care meet your expectations? Were you satisfied?
6. Can you describe a typical visit with the chiropractor? What is the nature of the treatment you receive?
7. Is there anything you would want done differently in your care?
8. Do you see other health care providers?
9. Does your chiropractor work with the other health care members that you see?
10. The profession is currently working on creating research topics and understanding you are not a researcher from your perspective as an athlete what areas should the profession investigate to explore areas of performance, injury, mechanics etc?
11. Would you recommend your chiropractor to other athletes that you compete with?
